# Exceptionally low likelihood of Alzheimer’s dementia in APOE2 homozygotes from a 5,000-person neuropathological study

**DOI:** 10.1038/s41467-019-14279-8

**Published:** 2020-02-03

**Authors:** Eric M. Reiman, Joseph F. Arboleda-Velasquez, Yakeel T. Quiroz, Matthew J. Huentelman, Thomas G. Beach, Richard J. Caselli, Yinghua Chen, Yi Su, Amanda J. Myers, John Hardy, Jean Paul Vonsattel, Steven G. Younkin, David A. Bennett, Philip L. De Jager, Eric B. Larson, Paul K. Crane, C. Dirk Keene, M. Ilyas Kamboh, Julia K. Kofler, Linda Duque, John R. Gilbert, Harry E. Gwirtsman, Joseph D. Buxbaum, Dennis W. Dickson, Matthew P. Frosch, Bernardino F. Ghetti, Kathryn L. Lunetta, Li-San Wang, Bradley T. Hyman, Walter A. Kukull, Tatiana Foroud, Jonathan L. Haines, Richard P. Mayeux, Margaret A. Pericak-Vance, Julie A. Schneider, John Q. Trojanowski, Lindsay A. Farrer, Gerard D. Schellenberg, Gary W. Beecham, Thomas J. Montine, Gyungah R. Jun, The Alzheimer’s Disease Genetics Consortium, Erin Abner, Perrie M. Adams, Marilyn S. Albert, Roger L. Albin, Liana G. Apostolova, Steven E. Arnold, Sanjay Asthana, Craig S. Atwood, Clinton T. Baldwin, Robert C. Barber, Lisa L. Barnes, Sandra Barral, James T. Becker, Duane Beekly, Eileen H. Bigio, Thomas D. Bird, Deborah Blacker, Bradley F. Boeve, James D. Bowen, Adam Boxer, James R. Burke, Jeffrey M. Burns, Nigel J. Cairns, Laura B. Cantwell, Chuanhai Cao, Chris S. Carlson, Cynthia M. Carlsson, Regina M. Carney, Minerva M. Carrasquillo, Helena C. Chui, David H. Cribbs, Elizabeth A. Crocco, Carlos Cruchaga, Charles DeCarli, Malcolm Dick, Rachelle S. Doody, Ranjan Duara, Nilufer Ertekin-Taner, Denis A. Evans, Kelley M. Faber, Thomas J. Fairchild, Kenneth B. Fallon, David W. Fardo, Martin R. Farlow, Steven Ferris, Douglas R. Galasko, Marla Gearing, Daniel H. Geschwind, Valentina Ghisays, Alison M. Goate, Neill R. Graff-Radford, Robert C. Green, John H. Growdon, Hakon Hakonarson, Ronald L. Hamilton, Kara L. Hamilton-Nelson, Lindy E. Harrell, Lawrence S. Honig, Ryan M. Huebinger, Christine M. Hulette, Gail P. Jarvik, Lee-Way Jin, Anna Karydas, Mindy J. Katz, John S. K. Kauwe, Jeffrey A. Kaye, Ronald Kim, Neil W. Kowall, Joel H. Kramer, Brian W. Kunkle, Amanda P. Kuzma, Frank M. LaFerla, James J. Lah, Yuk Ye Leung, James B. Leverenz, Allan I. Levey, Ge Li, Andrew P. Lieberman, Richard B. Lipton, Oscar L. Lopez, Constantine G. Lyketsos, John Malamon, Daniel C. Marson, Eden R. Martin, Frank Martiniuk, Deborah C. Mash, Eliezer Masliah, Wayne C. McCormick, Susan M. McCurry, Andrew N. McDavid, Stefan McDonough, Ann C. McKee, Marsel Mesulam, Bruce L. Miller, Carol A. Miller, Joshua W. Miller, John C. Morris, Shubhabrata Mukherjee, Adam C. Naj, Sid O’Bryant, John M. Olichney, Joseph E. Parisi, Henry L. Paulson, Elaine Peskind, Ronald C. Petersen, Aimee Pierce, Wayne W. Poon, Huntington Potter, Liming Qu, Joseph F. Quinn, Ashok Raj, Murray Raskind, Barry Reisberg, Joan S. Reisch, Christiane Reitz, John M. Ringman, Erik D. Roberson, Ekaterina Rogaeva, Howard J. Rosen, Roger N. Rosenberg, Donald R. Royall, Mark A. Sager, Mary Sano, Andrew J. Saykin, Lon S. Schneider, William W. Seeley, Amanda G. Smith, Joshua A. Sonnen, Salvatore Spina, Peter St George-Hyslop, Robert A. Stern, Russell H. Swerdlow, Rudolph E. Tanzi, Juan C. Troncoso, Debby W. Tsuang, Otto Valladares, Vivianna M. Van Deerlin, Linda J. Van Eldik, Badri N. Vardarajan, Harry V. Vinters, Sandra Weintraub, Kathleen A. Welsh-Bohmer, Kirk C. Wilhelmsen, Jennifer Williamson, Thomas S. Wingo, Randall L. Woltjer, Clinton B. Wright, Chuang-Kuo Wu, Chang-En Yu, Lei Yu, Yi Zhao

**Affiliations:** 1 Banner Alzheimer’s Institute and Arizona Alzheimer’s Consortium, 901 E Willetta Street, Phoenix, AZ 85006 USA; 2 University of Arizona, 714 E Van Buren Street, Phoenix, AZ 85006 USA; 3 Arizona State University, 522 N Central Avenue, Phoenix, AZ 85004 USA; 4 Neurogenomics Division, Translational Genomics Research Institute and Arizona Alzheimer’s Consortium, 445 N Fifth Street, Phoenix, AZ 85004 USA; 5 Schepens Eye Research Institute of Mass Eye and Ear and the Department of Ophthalmology at Harvard Medical School, 20 Staniford Street, Boston, MA 02114 USA; 6 Departments of Psychiatry and Neurology, Massachusetts General Hospital and Harvard Medical School, 55 Fruit Street, Boston, MA 02114 USA; 7 Mayo Clinic, 13400 E Shea Boulevard, Scottsdale, AZ 85259 USA; 8 Department of Psychiatry and Behavioral Science, University of Miami Miller School of Medicine, 1120 NW 14th Street, Miami, FL 33136 USA; 9 Department of Molecular Neuroscience, UCL, Institute of Neurology, Queen Square, London, WC1N 3BG UK; 10 New York Brain Bank and Department of Pathology, New York-Presbyterian Hospital at Columbia University Medical Center, 630 West 168th Street, New York, NY 10032 USA; 11 Departments of Neuroscience and Neurology, Mayo Clinic, 4500 San Pablo Road, Jacksonville, FL 32224 USA; 12 Departments of Neurological Sciences and Pathology (Neuropathology), and Rush Alzheimer’s Disease Center, Rush University Medical Center, 1725 W Harrison Street, Chicago, IL 60612 USA; 13 Department of Neurology, Center for Translational and Computational Neuroimmunology, Columbia University Medical Center, 710 West 168th Street, New York, NY 10032 USA; 14 Kaiser Permanente Washington Health Research Institute, 1730 Minor Avenue, Seattle, WA 98101 USA; 15 Department of Medicine, University of Washington, 1959 NE Pacific Street, Seattle, WA 98198 USA; 16 Department of Pathology, University of Washington, 1959 NE Pacific Street, Seattle, WA 98198 USA; 17 Department of Human Genetics, Alzheimer’s Disease Research Center, University of Pittsburgh, 200 Lothrop Street, Pittsburgh, PA 15213 USA; 18 Department of Pathology (Neuropathology), University of Pittsburgh, 200 Lothrop Street, Pittsburgh, PA 15213 USA; 19 Department of Neurology, University of Miami, 1120 NW 14th Street, Miami, FL 33136 USA; 20 John P. Hussman Institute for Human Genomics, Department of Human Genetics, and Dr. John T. Macdonald Foundation, University of Miami, 1501 NW 10th Avenue, Miami, FL 33136 USA; 21 Department of Psychiatry, Vanderbilt University, 1601 23rd Avenue South, Nashville, TN 37212 USA; 22 Departments of Psychiatry, Neuroscience and Genetics and Genomic Sciences, Mount Sinai School of Medicine, 1468 Madison Avenue, New York, NY 10029 USA; 23 Department of Neuroscience, Mayo Clinic, 4500 San Pablo Road, Jacksonville, FL 32224 USA; 24 C.S. Kubik Laboratory for Neuropathology, Massachusetts General Hospital, 114 16th Street, Charlestown, MA 02129 USA; 25 Department of Pathology and Laboratory Medicine, Indiana University, 340 West 10th Street, Indianapolis, IN 46202 USA; 26 Department of Biostatistics, Boston University School of Public Health, 801 Massachusetts Avenue, Boston, MA 02118 USA; 27 Department of Pathology and Laboratory Medicine, University of Pennsylvania Perelman School of Medicine, 3400 Spruce Street, Philadelphia, PA 19104 USA; 28 Department of Neurology, Massachusetts General Hospital and Harvard Medical School, 55 Fruit Street, Boston, MA 02114 USA; 29 Department of Epidemiology, University of Washington, 1959 NE Pacific Street, Seattle, WA 98195 USA; 30 Department of Medical and Molecular Genetics, Indiana University, 410W 10th Street, Indianapolis, IN 46202 USA; 31 Institute for Computational Biology and Department of Population and Quantitative Health Sciences, Case Western Reserve University, 10900 Euclid Avenue, Cleveland, OH 44106 USA; 32 Taub Institute on Alzheimer’s Disease and the Aging Brain, Gertrude H. Sergievsky Center Department of Neurology, Columbia University, 710 West 168th Street, New York, NY 10032 USA; 33 Biomedical Genetics Section, Department of Medicine, Boston University School of Medicine, 72 East Concord Street, Boston, MA 02118 USA; 34 Department of Neurology, Boston University School of Medicine, 72 East Concord Street, Boston, MA 02118 USA; 35 Department of Epidemiology, Boston University School of Public Health, 715 Albany Street, Boston, MA 02118 USA; 36 Department of Pathology, Stanford University, 300 Pasteur Drive, Stanford, CA 94305 USA; 37 Department of Epidemiology, College of Public Health, Sanders-Brown Center on Aging, University of Kentucky, Lexington, Kentucky USA; 38 Department of Psychiatry, University of Texas Southwestern Medical Center, Dallas, TX USA; 39 Department of Neurology, Johns Hopkins University, Baltimore, MD USA; 40 Department of Neurology, University of Michigan, Ann Arbor, MI USA; 41 Geriatric Research, Education and Clinical Center (GRECC), VA Ann Arbor Healthcare System (VAAAHS), Ann Arbor, MI USA; 42 Michigan Alzheimer Disease Center, Ann Arbor, MI USA; 43 Department of Radiology, Indiana University, Indianapolis, IA USA; 44 Department of Medical and Molecular Genetics, Indiana University, Indianapolis, IA USA; 45 Indian Alzheimer’s Disease Center, Indiana University, Indianapolis, IA USA; 46 Department of Neurology, Indiana University, Indianapolis, IA USA; 47 Department of Psychiatry and Behavioral Sciences, Duke University, Durham, NC USA; 48 Department of Psychiatry, University of Pennsylvania Perelman School of Medicine, Philadelphia, PA USA; 49 Geriatric Research, Education and Clinical Center (GRECC), University of Wisconsin, Madison, WI USA; 50 Department of Medicine, University of Wisconsin, Madison, WI USA; 51 Wisconsin Alzheimer’s Disease Research Center, Madison, WI USA; 52 Department of Pharmacology and Neuroscience, University of North Texas Health Science Center, Fort Worth, TX USA; 53 Departments of Psychiatry, Neurology, and Psychology, University of Pittsburgh School of Medicine, Pittsburgh, PA USA; 54 National Alzheimer’s Coordinating Center, University of Washington, Seattle, WA USA; 55 Department of Pathology, Northwestern University Feinberg School of Medicine, Chicago, IL USA; 56 Cognitive Neurology and Alzheimer’s Disease Center, Northwestern University Feinberg School of Medicine, Chicago, IL USA; 57 Department of Neurology, University of Washington, Seattle, WA USA; 58 VA Puget Sound Health Care System/GRECC, Seattle, WA USA; 59 Department of Epidemiology, Harvard School of Public Health, Boston, MA USA; 60 Department of Neurology, Mayo Clinic, Rochester, MN USA; 61 Swedish Medical Center, Seattle, WA USA; 62 Department of Neurology, University of California San Francisco, San Francisco, CA USA; 63 Department of Medicine, Duke University, Durham, NC USA; 64 University of Kansas Alzheimer’s Disease Center, University of Kansas Medical Center, Kansas City, KS USA; 65 Department of Pathology and Immunology, Washington University, St. Louis, MO USA; 66 USF Health Byrd Alzheimer’s Institute, University of South Florida, Tampa, FL USA; 67 Fred Hutchinson Cancer Research Center, Seattle, WA USA; 68 Department of Neurology, University of Southern California, Los Angeles, CA USA; 69 Department of Neurology, University of California Irvine, Irvine, CA USA; 70 Department of Psychiatry and Hope Center Program on Protein Aggregation and Neurodegeneration, Washington University School of Medicine, St. Louis, MO USA; 71 Department of Neurology, University of California Davis, Sacramento, CA USA; 72 Institute for Memory Impairments and Neurological Disorders, University of California Irvine, Irvine, CA USA; 73 Alzheimer’s Disease and Memory Disorders Center, Baylor College of Medicine, Houston, TX USA; 74 Wien Center for Alzheimer’s Disease and Memory Disorders, Mount Sinai Medical Center, Miami Beach, FL USA; 75 Department of Internal Medicine, Rush Institute for Healthy Aging, Rush University Medical Center, Chicago, IL USA; 76 Office of Strategy and Measurement, University of North Texas Health Science Center, Fort Worth, TX USA; 77 Department of Pathology, University of Alabama at Birmingham, Birmingham, AL USA; 78 Department of Biostatistics, Sanders-Brown Center on Aging, University of Kentucky, Lexington, KY USA; 79 Department of Neurology, Indiana University, Indianapolis, IN USA; 80 Department of Psychiatry, New York University, New York, NY USA; 81 Department of Neurosciences, University of California San Diego, La Jolla, CA USA; 82 Department of Pathology and Laboratory Medicine, Emory University, Atlanta, GA USA; 83 Emory Alzheimer’s Disease Center, Emory University, Atlanta, GA USA; 84 Neurogenetics Program, University of California Los Angeles, Los Angeles, CA USA; 85 Department of Medicine and Partners Center for Personalized Genetic Medicine, Division of Genetics, Brigham and Women’s Hospital and Harvard Medical School, Boston, MA USA; 86 Center for Applied Genomics, Children’s Hospital of Philadelphia, Philadelphia, PA USA; 87 Department of Neurology, University of Alabama at Birmingham, Birmingham, AL USA; 88 Department of Surgery, University of Texas Southwestern Medical Center, Dallas, TX USA; 89 Department of Pathology, Duke University, Durham, NC USA; 90 Department of Genome Sciences, University of Washington, Seattle, WA USA; 91 Department of Medicine (Medical Genetics), University of Washington, Seattle, WA USA; 92 Department of Pathology and Laboratory Medicine, University of California Davis, Sacramento, CA USA; 93 Department of Neurology, Albert Einstein College of Medicine, New York, NY USA; 94 Department of Biology, Brigham Young University, Provo, UT USA; 95 Department of Neurology, Oregon Health & Science University, Portland, OR USA; 96 Department of Neurology, Portland Veterans Affairs Medical Center, Portland, OR USA; 97 Department of Pathology and Laboratory Medicine, University of California Irvine, Irvine, CA USA; 98 Department of Pathology, Boston University, Boston, MA USA; 99 Department of Neuropsychology, University of California San Francisco, San Francisco, CA USA; 100 Department of Neurobiology and Behavior, University of California Irvine, Irvine, CA USA; 101 Department of Neurology, Emory University, Atlanta, GA USA; 102 Cleveland Clinic Lou Ruvo Center for Brain Health, Cleveland Clinic, Cleveland, OH USA; 103 Department of Psychiatry and Behavioral Sciences, University of Washington School of Medicine, Seattle, WA USA; 104 Department of Pathology, University of Michigan, Ann Arbor, MI USA; 105 Department of Psychiatry, Johns Hopkins University, Baltimore, MD USA; 106 Department of Medicine - Pulmonary, New York University, New York, NY USA; 107 Department of Pathology, University of California San Diego, La Jolla, CA USA; 108 School of Nursing Northwest Research Group on Aging, University of Washington, Seattle, WA USA; 109 PharmaTherapeutics Clinical Research, Pfizer Worldwide Research and Development, Cambridge, MA USA; 110 Department of Neurology, Northwestern University Feinberg School of Medicine, Chicago, IL USA; 111 Department of Pathology, University of Southern California, Los Angeles, CA USA; 112 Department of Neurology, Washington University, St. Louis, MO USA; 113 Internal Medicine, Division of Geriatrics, University of North Texas Health Science Center, Fort Worth, TX USA; 114 Department of Laboratory Medicine and Pathology, Mayo Clinic, Rochester, MN USA; 115 Michigan Alzheimer’s Disease Center, Department of Neurology, University of Michigan, Ann Arbor, MI USA; 116 Department of Neurology, University of Colorado School of Medicine, Aurora, CO USA; 117 Alzheimer’s Disease Center, New York University, New York, NY USA; 118 Department of Clinical Sciences, University of Texas Southwestern Medical Center, Dallas, TX USA; 119 Department of Epidemiology, Columbia University, New York, NY USA; 120 Tanz Centre for Research in Neurodegenerative Disease, University of Toronto, Toronto, ON Canada; 121 Department of Neurology, University of Texas Southwestern, Dallas, TX USA; 122 Departments of Psychiatry, Medicine, Family & Community Medicine, South Texas Veterans Health Administration Geriatric Research Education & Clinical Center (GRECC), UT Health Science Center at San Antonio, San Antonio, TX USA; 123 Department of Psychiatry, University of Southern California, Los Angeles, CA USA; 124 Cambridge Institute for Medical Research and Department of Clinical Neurosciences, University of Cambridge, Cambridge, UK; 125 Department of Pathology, Johns Hopkins University, Baltimore, MD USA; 126 Sanders-Brown Center on Aging, Department of Anatomy and Neurobiology, University of Kentucky, Lexington, KY USA; 127 Department of Neurology, University of California Los Angeles, Los Angeles, CA USA; 128 Department of Pathology and Laboratory Medicine, University of California Los Angeles, Los Angeles, CA USA; 129 Department of Genetics, University of North Carolina Chapel Hill, Chapel Hill, NC USA; 130 Department of Pathology, Oregon Health and Science University, Portland, OR USA; 131 Department of Neurology, Evelyn F. McKnight Brain Institute, Miller School of Medicine, University of Miami, Miami, FL USA; 132 Departments of Neurology, Pharmacology and Neuroscience, Texas Tech University Health Science Center, Lubbock, TX USA

**Keywords:** Alzheimer's disease, Genetics research

## Abstract

Each additional copy of the apolipoprotein E4 (APOE4) allele is associated with a higher risk of Alzheimer’s dementia, while the APOE2 allele is associated with a lower risk of Alzheimer’s dementia, it is not yet known whether APOE2 homozygotes have a particularly low risk. We generated Alzheimer’s dementia odds ratios and other findings in more than 5,000 clinically characterized and neuropathologically characterized Alzheimer’s dementia cases and controls. APOE2/2 was associated with a low Alzheimer’s dementia odds ratios compared to APOE2/3 and 3/3, and an exceptionally low odds ratio compared to APOE4/4, and the impact of APOE2 and APOE4 gene dose was significantly greater in the neuropathologically confirmed group than in more than 24,000 neuropathologically unconfirmed cases and controls. Finding and targeting the factors by which APOE and its variants influence Alzheimer’s disease could have a major impact on the understanding, treatment and prevention of the disease.

## Abstract (web summary)

APOE is the major genetic risk factor for Alzheimer’s disease. In a large number of neuropathologically confirmed cases and controls, the impact of different APOE genotypes on Alzheimer’s dementia risk was greater than previously thought and APOE2 homozygotes had an exceptionally low risk.

## Introduction

Apolipoprotein E (*APOE*), the major susceptibility gene for late-onset Alzheimer’s disease (AD), has three common alleles (APOE2, 3, and 4), giving rise to six genotypes (APOE2/2, 2/3, 3/3, 2/4, 3/4, and 4/4). Compared to the most common APOE3/3 genotype, each additional copy of the APOE4 allele is associated with a higher risk of Alzheimer’s dementia and a younger mean age at dementia onset, such that APOE4 homozygotes are at the highest risk, while the presence of one or two copies of the APOE2 allele is associated with a lower risk of Alzheimer’s dementia and an older mean age at dementia onset^1–4^. It remains to be clarified whether APOE2 homozygotes have a lower odd than persons with the APOE2/3 genotype—a question we sought to address in an unusually large number of clinically and neuropathologically classified Alzheimer’s dementia cases and controls.

We recently discovered two copies of the rare APOE3 Christchurch (APOE3ch [Arg136→Ser]) mutation, located in the ApoE protein’s low-density lipoprotein receptor (LDLR) binding region, in an amyloid-β_42_ (Aβ_42_)-overproducing presenilin 1 (PSEN1) E280A mutation carrier from the world’s largest autosomal dominant AD (ADAD) kindred who did not develop mild cognitive impairment (MCI) until her 70s, nearly three decades after her kindred’s mean age at MCI onset^5^. Using positron-emission tomography (PET) to compare her to other PSEN1 E280A mutation carriers with MCI, she had the greatest fibrillar amyloid-β (Aβ) burden (the major constituent of neuritic plaques), limited paired helical filament (PHF) tau (neurofibrillary tangle) burden, and minimal glucose hypometabolism in brain regions preferentially affected by AD. Like 5–10% of APOE2 homozygotes, she also had hyperlipoproteinemia Type III, reflecting reduced binding of the ApoE to LDLR^5,6^. Like the ApoE2 protein, the ApoEch protein was associated with less Aβ_42_ aggregation than the ApoE3 protein in vitro^5,7^. Since an APOE2 homozygote with an ADAD mutation did not have a significantly delayed clinical onset of ADAD, we postulated that homozygosity for APOEch may be more protective than homozygosity for APOE2, but that APOE2 homozygotes might still have an exceptionally low risk of late-onset Alzheimer’s dementia. We also postulated that APOE2 and APOE4 allelic dose would have a more profound impact on Alzheimer’s dementia odds ratios (ORs) in neuropathologically confirmed than in unconfirmed cases and controls due to the exclusion of clinically diagnosed cases who did not meet the neuropathological criteria for AD and unimpaired controls who met the criteria for AD, since misclassified cases and controls could dilute and/or bias OR estimates in the unconfirmed group.

This possibility has remained unaddressed, because case–control studies without neuropathological or biomarker assessments of AD may have underestimated the impact of APOE genotypes on Alzheimer’s dementia ORs due to the confounding effects of APOE genotypes on the percentages of neuropathologically misclassified cases and controls^8–10^. In fact, APOE4 non-carriers tend to be misclassified more frequently, such that 13% of APOE4 carriers versus 37% of non-carriers with the clinical diagnosis of mild-to-moderate Alzheimer’s dementia do not meet neuropathological criteria for AD^8^. Also, previous studies of clinically and neuropathologically characterized cases and controls may have been too small to demonstrate that APOE2 homozygotes have an even lower OR than the relatively low-risk APOE2/3 group due to the paucity of APOE2 homozygotes, who comprise <1% of the general population^1,4^.

This study sought to establish that APOE2 homozygotes have an exceptionally low likelihood of Alzheimer’s dementia, demonstrate the value of AD risk assessments in clinically and neuropathologically characterized cases and controls, and underscore the impact of different *APOE* genotypes on Alzheimer’s dementia ORs relative to the lowest risk APOE2/2 and highest risk APOE4/4 genotypes. More generally, it sought to highlight the impact of discovering and targeting the mechanism by which *APOE* variants account for differential risk could have on the understanding, treatment, and prevention of AD, including those interventions that might prevent both the initial development of AD pathology and the subsequent development of dementia.

## Results

### Neuropathologically confirmed and unconfirmed groups

Supplementary Table 1 shows the number of Alzheimer’s dementia cases and cognitively unimpaired controls for each APOE genotype in (a) the Alzheimer’s Disease Genetics Consortium (ADGC’s) clinically characterized and neuropathologically confirmed autopsy group, (b) its clinically characterized but neuropathologically unconfirmed clinical group, and (c) the combined neuropathological and clinical group. The 5007 participants in the neuropathologically confirmed cohort included 4018 AD dementia cases and 989 cognitively unimpaired and neuropathologically unaffected controls. The 23,857 participants in the clinically classified but neuropathologically unconfirmed cohort included 10,430 probable AD dementia cases and 13,426 cognitively unimpaired controls. The 28,864 participants in the combined group included 14,448 cases and 14,416 controls. Supplementary Table 2 summarizes ages at dementia onset in the cases, ages at last clinical exam in the cases, and ages at death in the neuropathologically confirmed autopsy cohort.

### Alzheimer’s dementia ORs

Table 1 and Supplementary Table 3 show Alzheimer’s dementia ORs for each APOE genotype and allelic doses (i.e., the number of APOE2 alleles in APOE4 non-carriers and the number of APOE4 alleles in APOE2 non-carriers) before and after adjustment for age and sex in the neuropathologically confirmed and unconfirmed groups before and after adjustment for age and sex, compared to the common APOE3/3 genotype. ORs associated with APOE2 allelic dose in APOE4 non-carriers (APOE2/2 < 2/3 < 3/3) and APOE4 allelic dose in APOE2 non-carriers (APOE4/4 > 3/4 > 3/3) were generated using allelic association tests in an additive genetic model. As discussed below, APOE2/2, APOE2/3, and APOE2 allelic dose ORs were significantly lower, and APOE3/4, APOE4/4, and APOE4 allelic dose ORs were significantly higher, in the neuropathologically confirmed group than in the unconfirmed group. While ORs for the other APOE genotypes were similar to those that we had reported in a small number of cases and controls, the number of APOE2 homozygotes in the earlier study was too small to provide an accurate OR estimate^1,11^. Table 2 shows Alzheimer’s dementia ORs for each APOE genotype compared to the relatively low-risk APOE2/3 and highest risk APOE4 genotypes in the neuropathologically confirmed cohort. As discussed below, these ORs permitted us to confirm our primary hypothesis that APOE2/2 is associated with a significantly lower OR compared to APOE3/3 and to demonstrate an exceptionally low OR compared to APOE4/4. Supplementary Table 4 shows Alzheimer’s dementia ORs for each APOE genotype in the combined group, compared to APOE3/3, and for APOE2 and APOE4 allelic dose before and after adjustment for age, sex, and autopsy/non-autopsy group.

**Table 1:** Association of APOE genotypes and allelic doses compared to the APOE3/3 genotype.

APOE	Neuropathologically confirmed group			Neuropathologically unconfirmed group		
	OR	95% CI	*P*	OR	95% CI	*P*
Genotype						
2/2	0.13	0.05–0.36	6.3 × 10^−5^	0.52	0.30–0.90	0.02
2/3	0.39	0.30–0.50	1.6 × 10^−12^	0.63	0.53–0.75	2.2 × 10^−7^
2/4	2.68	1.65–4.36	7.5 × 10^−5^	2.47	2.02–3.01	5.7 × 10^−19^
3/4	6.13	5.08–7.41	2.2 × 10^−75^	3.55	3.17–3.98	2.3 × 10^−105^
4/4	31.22	16.59–58.75	4.9 × 10^−26^	10.70	9.12–12.56	7.5 × 10^−186^
Allelic dose						
2	0.38	0.30–0.48	1.1 × 10^−15^	0.64	0.58–0.72	2.2 × 10^−16^
4	6.00	5.06–7.12	3.4 × 10^−90^	3.43	3.26–3.60	<10^−300^

For genotypic association tests, odds ratio (OR), 95% confidence interval (CI), and *P* value (*P*) for each APOE genotype compared to the APOE3/3 genotype were calculated under a logistic regression model.

For allelic association tests, OR, CI, and *P* associated with APOE2 allelic dose in APOE4 non-carriers (APOE2/2 < 2/3 < 3/3) and APOE4 allelic dose in APOE2 non-carriers (APOE4/4 > 3/4 > 3/3) in an additive genetic model were generated under a logistic regression model.

**Table 2:** Association of each APOE genotype in the neuropathologically confirmed group.

APOE	Compared to APOE2/3			Compared to APOE4/4		
	OR	95% CI	*P*	OR	95% CI	*P*
2/2	0.34	0.12–0.95	0.04	0.004	0.001–0.014	6.0 × 10^−19^
2/3	Ref.	Ref.	Ref.	0.012	0.006–0.024	1.2 × 10^−34^
3/3	2.60	2.00–3.38	1.6 × 10^−12^	0.032	0.017–0.060	4.9 × 10^−26^
2/4	6.96	4.06–11.92	7.5 × 10^−12^	0.086	0.039–0.189	1.6 × 10^−9^
3/4	15.92	11.85–21.38	1.4 × 10^−70^	0.196	0.103–0.375	8.4 × 10^−7^
4/4	81.05	41.39–158.68	1.2 × 10^−34^	Ref.	Ref.	Ref.

Alzheimer’s dementia odds ratios (ORs), 95% confidence intervals (CIs), and *P* value (*P*) for each APOE genotype compared to the APOE2/3 or 4/4 genotype as a reference (Ref.) in the neuropathologically confirmed group were calculated under a logistic regression model.

As shown in Tables 1 and 2, APOE2 homozygotes in the neuropathologically confirmed group had a significantly lower 0.34 OR (95% confidence interval (CI) = 0.12–0.95) compared to the relatively low-risk APOE2/3 genotype*,* an extremely low 0.13 OR (95% CI = 0.05–0.36) compared to the most common APOE3/3 genotype; and an exceptionally low 0.004 OR (95% CI = 0.001–0.014) compared to the highest risk APOE4/4 genotype. As shown in Supplementary Tables 3 and 4, APOE2 homozygotes had more modest, but significantly lower ORs compared to APOE3/3 in both the clinical group (0.52 OR; 95% CI = 0.30–0.90) and the combined group (0.43 OR; 95% CI = 0.26–0.70), while ORs in the neuropathologically confirmed, unconfirmed, or combined group were not significantly affected after adjustment for age and sex.

As shown in Table 1*,* APOE2 allelic dose in APOE4 non-carriers was associated with significantly lower ORs in both the neuropathologically confirmed and unconfirmed groups, and with significantly lower ORs in the confirmed group than in the unconfirmed group (Table 1). As shown in Supplementary Tables 3 and 4, ORs in the neuropathologically confirmed (0.38 OR; 95% CI = 0.30–0.48; *P* = 1 × 10^−15^), unconfirmed (0.64 OR; 95% CI = 0.58–0.72; *P* = 2 × 10^−16^), and combined groups (0.59 OR; 95% CI = 0.53–0.65; *P* = 9 × 10^−27^) were similar to the corresponding APOE2/3 ORs relative to APOE3/3. These findings were not significantly affected after adjustment for age, sex, and neuropathologically confirmed autopsy/unconfirmed non-autopsy group.

APOE2/4, APOE3/4, and APOE4/4 genotypes compared to the most common APOE3/3 genotype in the neuropathologically confirmed autopsy group were associated with 2.68, 6.13, and 31.22 ORs, respectively. These APOE4 carrying genotypes compared to the lowest risk APOE2/2 genotype increased ORs with 20.33, 46.51, and 236.74, respectively, while, compared to the highest odds APOE4/4 genotype decreased ORs with 0.09, 0.20, and 1.00, respectively. These results underscore the impact of *APOE* and its common *APOE* genotypes on the differential associations of Alzheimer’s dementia, the progressively harmful or protective molecular mechanisms that may account for these differences, and the importance of discovering interventions to safely and sufficiently target those factors to the treatment and prevention of AD. As we predicted from the likely inclusion of neuropathologically misclassified cases and controls in the clinical group, APOE3/4 and 4/4 ORs were significantly lower than those in the neuropathologically confirmed autopsy group and roughly comparable to those from numerous case–control studies in which neuropathological (or biomarker) measurements were not required to confirm the presence or absence of AD^1^. APOE2/4, 3/4, and 4/4 Alzheimer’s dementia ORs relative to APOE3/3 were 2.47, 3.55, and 10.70, in the neuropathologically unconfirmed group (Table 1), and 2.47, 3.78, and 12.02 in the combined group (Supplementary Table 4), respectively.

As shown in Table 1 and Supplementary Tables 3 and 4, APOE4 allelic dose in APOE2 non-carriers was associated with significantly greater ORs in the neuropathologically confirmed autopsy, neuropathologically unconfirmed clinical, and combined groups. Using an additive genetic model, ORs in the neuropathologically confirmed (6.00 OR; 95% CI = 5.06–7.12; *P* = 3 × 10^−90^), unconfirmed (3.43 OR; 95% CI = 3.26–3.60; *P* < 1 × 10^−300^), and combined group (3.61 OR; 95% CI = 3.37–3.87; *P* = 2 × 10^−290^) were similar to corresponding APOE3/4 OR relative to APOE3/3. As shown in Supplementary Tables 3 and 4, findings were not significantly different after adjustment for age or sex in the neuropathologically confirmed or unconfirmed group or after adjustment for age, sex, or neuropathologically confirmed autopsy/unconfirmed non-autopsy group.

The receiver operating characteristic (ROC) curves in Supplementary Fig. 1 show the impact of APOE2 and APOE4 allelic doses on the classification of cases and controls in the neuropathologically confirmed autopsy and unconfirmed non-autopsy groups. APOE2 and APOE4 allelic doses were each associated with significantly greater areas under the curve (AUC), an indicator of classification accuracy, in the neuropathologically confirmed and unconfirmed groups, and APOE4 allelic dose had a greater impact than APOE2 allelic dose on AUCs in both groups. While APOE2 allelic dose was associated with a significantly greater AUC in the neuropathologically confirmed autopsy than in the unconfirmed non-autopsy group (AUC 0.68 [95% CI: 0.65–70] versus 0.58 [95% CI: 0.50–0.65]), corresponding to variance importance scores of 24.0 versus 9.0, respectively, AUCs for APOE4 allelic dose were not significantly different in the neuropathologically confirmed autopsy and non-autopsy groups, as reflected by AUC 0.77 (95% CI: 0.75–79) versus 0.75 (95% CI: 0.74–0.77) and corresponding variance importance scores of 43.9 and 32.3.

### ORs for four other neuropathological diseases

Other neuropathological diseases are relatively common in older adults with and without Alzheimer’s dementia. Supplementary Table 5 shows the number of persons in the neuropathologically confirmed case–control group with and without four commonly assessed neuropathological diagnoses, including congophilic amyloid angiopathy (CAA), Lewy body disease (LBD), vascular brain injury (VBI), and hippocampal sclerosis (HS). (Since TDP-43 pathology and microinfarcts were not characterized in many of the participants, they were not included in our analysis.) CAA, LBD, VBI, and HS were present in 94%, 88%, 78%, and 83% of the Alzheimer’s dementia cases and 70%, 22%, 12%, and 14% of the unimpaired non-AD controls. Supplementary Tables 6 and 7 show CAA, LBD, VBI, and HS ORs for each APOE genotype, compared to APOE3/3, and for allelic dose, before and after adjustment for age, sex, and the neuropathological diagnosis of AD. APOE2 allelic dose was not associated with a significantly lower OR for any of these diseases, before or after adjustment for the presence or absence of AD. While APOE4 allelic dose was not significantly associated with significantly higher VBI and HS ORs, it was associated with significantly higher CAA and LBD ORs, before and after adjustment for age, sex, and presence or absence of AD.

### Ages at dementia onset

**Fig. 1:**
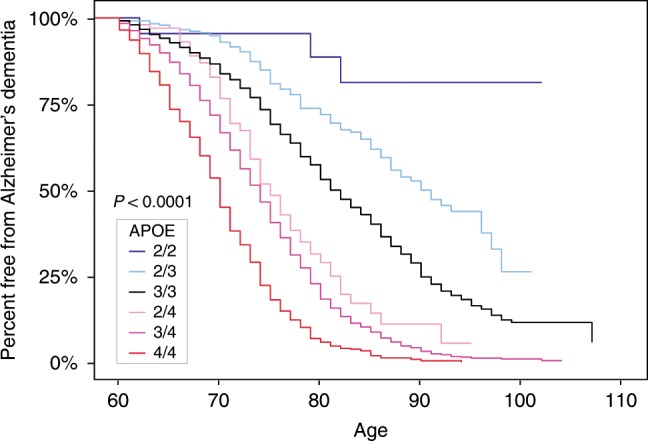
Percent free from Alzheimer’s dementia. Kaplan–Meier curves were generated from Alzheimer’s dementia cases and cognitively unimpaired non-AD controls in the neuropathologically confirmed group. *Y*-axis represents the percentage of persons with each APOE genotype in the neuropathologically confirmed group who remained free from Alzheimer’s dementia. *X*-axis denotes age at death for controls and age at onset of cases, while replacing with age at death when age at onset was unavailable.

The estimated mean ages at Alzheimer’s dementia onset for each genotype shown in Supplementary Table 2 are consistent with previously reported findings. In neuropathologically confirmed Alzheimer’s dementia cases with available onset ages, APOE4/4, 3/4, 2/4, 3/3, and combined 2/3 and 2/2 genotypes were associated with progressively older ages at Alzheimer’s dementia onset, ranging from 69.9 ± 6.1 years in the APOE4/4 genotype to 79.3 ± 9.0 years in the combined APOE2/3 and 2/2 group. In the neuropathologically unconfirmed and combined cases, APOE4/4, 3/4, 2/4, 3/3, and combined 2/3 and 2/2 genotypes were also associated with progressively older ages at Alzheimer’s dementia onset, ranging from 69.5 ± 5.9 years in the combined APOE4/4 homozygote group to 77.7 ± 8.5 years in the combined APOE2/3 and 2/2 group. We used age at death as a proxy age when age at onset in cases were not available. The Kaplan–Meier curves in Fig. 1 show the percentage of persons in the neuropathologically confirmed case–control group with each APOE genotype, including APOE2/2, who remained free from Alzheimer’s dementia at different ages. While there was some overlap between the 95% CIs in the APOE2/2 and 2/3 plots due to the small size of and relatively large CI for the APOE2/2 group, the Kaplan–Meier plots in Fig. 1 confirmed a relationship between APOE2 allelic dose and freedom from Alzheimer’s dementia survival at older ages.

### Neuritic plaque and neurofibrillary tangle severity

Supplementary Table 8 and Supplementary Fig. 2 provide information about Consortium to Establish a Registry (CERAD) (neuritic Aβ plaque) scores and Braak (tau tangle) stage in the neuropathologically verified case–control group, which contained only neuropathologically confirmed AD cases and pathology-free controls. APOE genotype (APOE2/2 < 2/3 < 3/3 < 2/4 < 3/4 < 4/4) (and consequently allelic dose) were associated with greater Aβ plaque and tau tangle severity, before and after adjustment for age at death and sex. Our findings could be attributable to the proportion of cases to controls in each genetic group, their direct or indirect impact on these neuropathological features, potentially confounding differential effects of AD onset and duration on Braak stage, or a combination of these and other factors. While the difference between CERAD scores in the small APOE2/2 and 2/3 groups were not significant (1.05 ± 1.10 versus 1.33 ± 1.31, *P* = 0.35), APOE2 homozygotes were distinguished from the APOE2/3 group by a significantly lower Braak stage (2.26 ± 1.63 versus 3.16 ± 1.69, *P* < 0.05), before and after adjustment for age and sex. Compared to the APOE3/3 group, the APOE2/2 and APOE2/3 groups had significantly lower CERAD scores and Braak stages, and the APOE2/4, 3/4, and 4/4 genotypes had significantly higher CERAD scores and Braak stages, before and after adjustment for age at death and sex.

Residual effects of each APOE genotype on Braak stage, relative to APOE2/2, 3/3, and 4/4, after controlling CERAD scores are depicted in terms of *β* (linear regression) coefficients in Table 3. Relatively protective or harmful effects are reflected by negative or positive *β* coefficients, respectively. Compared to APOE2/3 or APOE3/3, only the APOE3/4 and 4/4 genotypes demonstrated progressively harmful residual effects on Braak stage. Compared to APOE4/4, the APOE2/2, 2/3, 3/3, 2/4, and 3/4 genotypes had significant and progressively protective residual effects on Braak stage with *β* coefficients of −1.32, −0.43, −0.25, −0.26, and −0.10, respectively (*P* < 0.05), such that the protective effect was more than three times greater in the APOE2/2 group than in the APOE2/3 group. This finding supports the possibility that APOE variants have differential effects on tau tangle severity, even after controlling for neuritic Aβ plaque severity.

**Table 3:** Residual effects of Braak stage after adjustment for plaque core.

*APOE*	Compared to APOE2/3			Compared to APOE3/3			Compared to APOE4/4		
	BETA	SE	*P*	BETA	SE	*P*	BETA	SE	*P*
2/2	−0.44	0.31	0.15	−0.50	0.28	0.07	−1.32	0.27	1.6 × 10^−6^
2/3	Ref.	Ref.	Ref.	−0.07	0.09	0.44	−0.43	0.11	1.2 × 10^−4^
3/3	0.07	0.09	0.39	Ref.	Ref.	Ref.	−0.25	0.06	4.3 × 10^−5^
2/4	0.13	0.16	0.40	0.03	0.12	0.80	−0.26	0.11	0.02
3/4	0.29	0.08	6.1 × 10^−4^	0.15	0.04	1.9 × 10^−4^	−0.10	0.05	0.05
4/4	0.42	0.10	7.5 × 10^−5^	0.25	0.06	4.3 × 10^−5^	Ref.	Ref.	Ref.

*β* estimate (BETA), standard error (SE), and *P* (*P* value) for each *APOE* genotype compared to the APOE2/3, 3/3, and 4/4 genotype as a reference (Ref.) were calculated under a linear regression model; NA: not applicable. *β* estimates reflect the impact of each *APOE* genotype on Braak (tau tangle) stage after adjustment for CERAD (neuritic Aβ plaque) score.

## Discussion

This study demonstrates an exceptionally low likelihood of Alzheimer’s dementia in APOE2 homozygotes in a large group of clinically and neuropathologically characterized cases and controls. The large number of cases and controls in the neuropathologically unconfirmed and combined groups enabled us to demonstrate a significantly greater impact of APOE2/2, 2/3, 3/3, 3/4, and 4/4 genotypes on Alzheimer’s dementia ORs in the neuropathologically confirmed group—and to suggest that the greater impact may be attributable to the exclusion of cases without significant AD neuropathology, as well as the exclusion of controls with preclinical AD neuropathology^12^. This study provides updated ORs for Alzheimer’s dementia for each of the six common APOE genotypes, APOE2 allelic dose, and APOE4 allelic dose on, on the differential risk of Alzheimer’s dementia, free from the confounding effects of clinically misdiagnosed cases and controls, and it demonstrates an additional impact of APOE4, but not APOE2 allelic dose on two other neuropathological disease (CAA and DLB) ORs. The study supports known effects of *APOE* genotypes on standard measures of neuritic Aβ plaque and tau tangle severity^13^ and suggests progressively protective residual effects on Braak stage in APOE3/4, 2/4, 3/3, 2/3, and 2/2 groups compared to APOE4/4 homozygotes.

The APOE2/2 genotype was associated with a significantly lower 0.34 Alzheimer’s dementia OR compared to the relatively low odds APOE2/3 genotype (95% CI: 0.12–0.95), an extremely low 0.13 OR compared to the most common APOE3/3 genotype (95% CI: 0.05–0.36), and an exceptionally low 0.004 OR compared to the highest odds APOE4/4 genotype (95% CI: 0.001–0.014) in those neuropathologically confirmed subjects. In other words, persons with the APOE2/2 genotype had a 66% lower OR than those with the APOE2/3 genotype, 87% lower than those with the APOE3/3 genotype, and 99.6% (95% CI: 98.6–99.9%) lower than those with APOE4/4 group. These findings highlight the impact of *APOE* and its variants on the risk of AD and the potential impact of *APOE*-modifying interventions on its treatment and prevention.

The APOE2/2 genotype has recently been suggested to be associated with more severe pathology in primary tauopathies, including progressive supranuclear palsy, corticobasal degeneration, and a mouse model of human tau over-expression^14^. On the other hand, APOE2/2 and APOE3/3ch genotypes could be associated with less severe tau pathology in AD (a secondary tauopathy) due to a direct or indirect (e.g., amyloid and neuroinflammation-mediated) effects, as noted above. While the current study did not assess the impact of APOE2 allelic dose on ORs for these primary tauopathies, it found no association between APOE2 allele dose and ORs for four other disease (CAA, DLB, VBI, and HS). In contrast, APOE4 allelic dose was associated with significantly higher ORs for CAA and DLB, before and after adjustment for the presence or absence of AD, and not with VBI or HS^11^. These findings are similar to those described in a study involving many of these cases and controls, even though we did not include persons with the APOE3/4 genotype in our assessment of APOE2 and APOE4 allelic doses.

As previously noted, we and our colleagues recently found as association between two copies of the rare APOE3ch mutation and resistance to the clinical onset of AD in an Aβ_42_-overproducing PSEN1 E280A mutation carrier from the world’s largest ADAD kindred^5^. Interestingly, this individual had unusually high PET measurements of Aβ plaque burden, relatively limited PET measurements of PHF tau (neurofibrillary tangle) burden, minimal cerebral glucose hypometabolism in AD-affected brain regions, and like some APOE2 homozygotes, Type III hyperlipoproteinemia^5,6^. Associations between APOE variants and AD (APOE4 > 3 > 2 and APOE3ch) and other findings led us to postulate that the contributions of *APOE* and its variants to the differential risk of Alzheimer’s dementia are not solely attributable to their impact on the density of Aβ plaques but also to their direct or indirect impact on downstream pathogenic events. In experimental studies, we found that the ApoEch protein was associated with reduced Aβ_42_ burden and a differential impact of ApoE isoforms (ApoE4 > 3 > 2 >> 3ch) on heparin binding, and demonstrated the ability of a targeted antibody to lower wild-type ApoE3 binding^15^. Based on studies implicating heparin sulfate polyglycan (HSPG) on Aβ aggregation, Aβ-mediated microglial response, and the neuronal uptake and propagation of tau, and neurodegeneration, we postulated that the ApoE binding to HSPG could have potential roles in the pathogenesis, treatment, and prevention of AD^16^.

Together, our APOE2/2 and APOE3ch/3ch studies underscore the need to clarify and target the factors by which APOE and its variants account for this differential risk, treatment, and prevention of AD. It remains to be shown whether the differential effects of *APOE* variants on the risk of Alzheimer’s dementia are related to their recognized effects on Aβ oligomerization^5–7^, morphology, or clearance, their suggested effects on *TREM2*-mediated microglial response, tau pathology, LDLR binding, HSPG binding, mitochondrial function, neurodegeneration, a non-additive protective effect of APOE2 in reducing the expression of a microglial aging signature (HuMi Aged geneset)^17^, or a combination of these and other effects^5,7,18–23^. It also remains to be shown whether it would help to increase or, as some of us suggest, decrease ApoE expression in the brain, and whether ApoEch and ApoE2 isoforms might be associated with a relative loss in one or more functions that are critically involved in the development of AD. If so, *APOE* and its variants could differ in the extent of their pathogenic functions (APOE4/4 > 3/4 > 2/4 > 3/3 > 2/3 > 2/2 > APOEch/ch > no APOE)^5^. We postulate that genetic, drug, or immune treatments that safely and sufficiently inhibit the expression of *APOE* or its relevant functions in brain might have a significant impact on the treatment and prevention of AD. Evidence from a person without *APOE* function due to homozygosity for an ablative *APOE* frameshift mutation^24^ and the availability of dyslipidemia treatments support the potential tolerability of this approach. Additional research is needed to clarify the mechanisms by which homozygosity for APOE2 and APOEch are associated with an exceptionally low risk of AD dementia. Gene editing, protein-reducing, protein-modifying, or other treatments that safely and sufficiently replicate protective effects of the APOE2/2 genotypes could help to prevent the clinical onset of AD.

The study has several limitations. First, while the unusually large number of participants in the overall study, there was a relatively small number of APOE2 homozygotes. Despite the resulting limitation in statistical power, it did not prevent us from demonstrating significant effects in association with the APOE2/2 genotype and APOE2 allelic dose. Second, as previously noted, since we do not have information from most of the participating brain banks’ neuropathologically misclassified cases and controls, we are not able to clarify with the more profound impact of different APOE genotypes and allelic doses on Alzheimer’s dementia ORs is solely attributable to the exclusion of neuropathologically misclassified cases and controls or also to any ascertain biases related to participation in a brain donation program. However, we were able to quantify and distinguish the impact of APOE2 versus APOE4 allelic doses on the classification of cases and controls in both the neuropathologically confirmed and unconfirmed groups. Third, we cannot exclude an impact of differential disease onset, duration, or survival on Alzheimer’s dementia ORs and measures of AD pathology. Fourth, findings from our non-Hispanic White groups cannot yet be extended to other ethnic and racial groups^4^.

Prospective cohort studies that include persons irrespective of their cognitive stage or neuropathological diagnosis are needed to clarify the absolute lifetime risk of neuropathologically confirmed Alzheimer’s dementia for each *APOE* genotype. Similarly, neuropathological studies that include brain donors irrespective of their cognitive stage or neuropathological diagnosis are needed to further clarify the impact of APOE2 gene dose on tau pathology and neurodegeneration, including the extent to which this impact is or is not mediated through its effect on Aβ pathology^8,25^. Since the reported prevalence and impact of different APOE genotypes on Alzheimer’s dementia risk depends in part on age, race, ethnicity, geographic location, education, and dementia severity, these estimates are likely to vary in different populations. Based on our selection criteria, this study does not provide information about the percentage of *APOE* genotypes in cognitively unimpaired persons with neuropathological or biomarker evidence of preclinical AD, the percentage of persons who met criteria for MCI with or without neuropathological or biomarker evidence of AD, or the percentage of persons with a primary diagnosis of other neurodegenerative disorders. The impact of different APOE genotypes on estimated ages at Alzheimer’s dementia onset may have been greater if standardized prospective assessments had been used to estimate onset ages at every site, if the study included more research participants who developed Alzheimer’s dementia at younger ages (e.g., preferentially reducing onset ages in the APOE4 carrier groups), and if it included more participants who developed Alzheimer’s dementia at the oldest ages, for example, preferentially increasing onset ages in the APOE4 non-carrier groups.

This study supports the complementary value of risk factor assessments in large neuropathologically confirmed autopsy and even larger neuropathologically unconfirmed clinical groups. In a previous autopsy study, we found that 25% of persons with the clinical diagnosis of mild-to-moderate Alzheimer’s dementia lacked at least moderately frequent neuritic plaques, one of the cardinal features of AD, including 37% of APOE4 non-carriers and 13% of carriers, findings that are consistent with those in living patients^9,10^. In a meta-analysis of brain imaging studies, about 25% of cognitively unimpaired older adults have brain imaging evidence consistent with at least moderately frequent plaques. By investigating clinically characterized cases who are confirmed to have AD to unimpaired controls who are confirmed to be free of AD, it may be possible to address potentially confounding effects of the risk factor on the presence or absence of AD and underscore the potential impact of an intervention to prevent both AD and its clinical consequences. By investigating an even larger number of cases and controls without biological confirmation, it may be possible to clarify risk factors without potentially confounding effects on the underlying disease with improved statistical power and greater generalizability to understudied populations. Brain imaging and fluid biomarkers have the potential increase the size and generalizability of findings in case–control studies of AD.

Additional research is needed to clarify the mechanism by which *APOE* and its variants contribute to the pathogenesis and potential treatment and prevention of AD. There is a critical need to discover treatments that account for impact of genotypes on the differential risk of Alzheimer’s dementia, including those that may account for a profound resistance to Alzheimer’s dementia in APOE2 and APOEch homozygotes, and to establish their value in the treatment and prevention of AD.

In conclusion, homozygosity for the APOE2 allele appears to be associated with an exceptionally low likelihood of AD dementia, *APOE* genotypes have an important impact on Alzheimer’s dementia ORs, and treatments that target *APOE* and its variants could have an important impact on the treatment and prevention of the disease.

## Methods

### Subjects

Our primary analysis capitalized on data from 5007 brain donors in the ADGC’s neuropathologically confirmed autopsy group (confirmed group), including 4018 cases who met the clinical and neuropathological criteria for Alzheimer’s dementia and 989 cognitively unimpaired controls who did not meet the neuropathological criteria for AD were described in prior reports; 5 of the cases and 19 of the controls had the APOE2/2 genotype. There were 283 brain donors in the ADGC’s “neuropathologically misclassified autopsy group,” including 123 cases who met the clinical criteria for probable Alzheimer’s dementia, but did not meet the neuropathological criteria for AD, and 160 unimpaired controls who met the neuropathological criteria for AD. The entire autopsy group consisted of unrelated cases and controls. The ADGC’s neuropathologically unconfirmed clinical group (unconfirmed group) contained 23,857 living research participants, including 10,430 cases who met the clinical criteria for probable Alzheimer’s dementia and 13,427 cognitively unimpaired controls, and the combined (neuropathologically confirmed and unconfirmed) group contained 28,864 research participants, including 14,448 cases and 14,416 controls. Data from the neuropathologically confirmed autopsy group were used in our primary analyses; data from the other groups were in post hoc comparisons with that in the autopsy group. Due to the prioritized ascertainment of those cases who met the clinical and neuropathological criteria for Alzheimer’s dementia and those unimpaired controls without AD, the number of misclassified cases and controls available through the AD Genetics Consortium^12^ is much smaller than our estimated number of neuropathologically misclassified cases and controls^8–11^, limiting our ability to clarify the impact of brain donation on APOE ORs in our post hoc analyses. The number of cases and controls for each APOE genotype in the ADGC’s neuropathologically confirmed autopsy group, neuropathologically unconfirmed clinical group, and combined confirmed and unconfirmed group is shown in Supplementary Table 1.

### Phenotypic evaluation

Brain samples, extracted DNA, and demographic, clinical, and neuropathological data from clinically and neuropathologically characterized brain donors were assembled by the ADGC in conjunction with past and present National Institute on Aging (NIA)-sponsored AD Centers, Banner Sun Health Research Institute and the Translational Genomics Research Institute (TGen), the Adult Changes in Thought Study (ACT), the University of Miami’s Brain Endowment Bank and Hussman Institute for Human Genomics, the Late-onset Alzheimer’s Disease Family Study, the Religious Orders Study and Memory Aging Project (ROS-MAP), the Vanderbilt University Center for Human Genetics Research, and the National Alzheimer’s Coordinating Center; *APOE* genotypes were characterized at the National Cellular Repository for AD or TGen. Neuritic plaque burden was scored using the CERAD 0–3 point (none, sparse, moderate, or frequent plaque) rating system, the spatial extent of neurofibrillary tangle (PHF tau) burden was scored using the Braak 0–VI staging system, and neuropathological data were reviewed and harmonized by a single neuropathologist (Thomas Montine)^11,13^. Neuropathological data in the autopsy group was collected at each site according to consensus guidelines at the time of brain autopsy. Cases with neuropathologic evidence of disease other than AD neuropathologic change, with or without common co-morbid lesions, were excluded. CERAD score, which provide an indicator of neuritic (Aβ) plaque severity, and Braak stage, which provides an indicator of neurofibrillary (tau) tangle spatial extent and severity, were available in every participant in the ADGC’s neuropathologically confirmed case–control autopsy group. Data regarding the presence or absence of four other neuropathological diagnoses commonly found in persons with AD, including CAA, LBD, VBI, and HS, was most of the participants. Since TDP-43 proteinopathy and microinfarcts were not available in most of the brain donors (many of whom came to autopsy prior to development of TDP-43 proteinopathy), these neuropathological diseases were not included in our analysis. CAA, LBD, VBI, and HS were present in 84%, 43%, 38%, and 21% of the Alzheimer’s dementia cases and unimpaired non-AD controls.

The Alzheimer’s dementia cases met the DSM-IV or NINCDS/ADRDA criteria for dementia^26^ and, when available, had Clinical Diagnostic Ratings (CDRs) greater than zero before they died^27^; they either met the NIA/Reagan neuropathological criteria for intermediate-to-high likelihood AD or had both moderate-to-frequent neuritic plaque scores (i.e., CERAD score 2–3) and spatially extensive neurofibrillary tangle burden (i.e., Braak stage III–VI)^28–30^. The controls did not meet the clinical criteria for dementia or MCI and, when available, had a CDR of zero, within 2 years before they died; they met the neuropathological criteria for low-likelihood AD, had sparse neuritic plaques (CERAD score 1) and spatially limited tangle burden (Braak stages 0–II), or had no neuritic plaques (CERAD score 0) and no more than moderately extensive tangle burden (Braak stages 0–IV).

For comparative purposes, we subsequently analyzed *APOE* and other relevant data in clinically diagnosed but neuropathologically unexamined participants assembled by the ADGC from non-Hispanic whites. Ages at clinical onset (when available), last clinical examination, and age at death of the ADGC sample^31^ are shown for each *APOE* genotype in Supplementary Tables 2 and 3.

### Statistical analysis

For the assessment of Alzheimer’s dementia ORs associated with each *APOE* genotype, we coded the *APOE* genotype of interest as 1, coded the reference genotype as 0 (APOE2/2, APOE3/3, or APOE4/4), and treated other genotypes as missing. We used clinical diagnosis as a binary outcome (AD) for the neuropathologically confirmed, clinical, and combined group. ORs and 95% CIs for each *APOE* genotype were computed compared to a reference *APOE* genotype. We conducted genotypic association tests for each *APOE* genotype compared to a reference genotype used for AD, CAA, LBD, VBI, and HS with and without covariate adjustment for age and sex. For the neuropathologically confirmed group, we computed ORs under a logistic regression using a generalized linear model (GLM). For clinical and combined groups, we further accounted for family structure due to containing families from the Multi-Institutional Research on Alzheimer’s Genetic Epidemiology and NIA Late-onset AD (NIA-LOAD) study^32^ under a logistic regression using generalized estimating equations. For the assessment of APOE2 allelic dose treating APOE2 or APOE4 allele as a bi-allelic genetic variant, we coded APOE3/3, APOE2/3, and APOE2/2 as 0, 1, 2, respectively, in the APOE4 non-carriers; and for the assessment of APOE4 allelic dose, we coded APOE3/3, APOE3/4, and APOE4/4 as 0, 1, 2, respectively, in the APOE2 non-carriers. We conducted allelic association tests for the APOE2 and APOE4 under a logistic regression using a GLM for AD, CAA, LBD, VBI, and HS with and without covariate adjustment for age and sex. We further adjusted for AD in four other neuropathological diagnoses, CAA, LBD, VBI, and HS We used a sensitivity analysis, ROC curves, and AUCs to characterize and compare the contribution of APOE2 and APOE4 allelic doses on the classification of cases and controls in the neuropathologically confirmed and unconfirmed groups. To rank predictors based on their contributions to the logistic regression model, we calculated variance importance score using the *varImp* option in R, which the importance score is ranged from 0 to 100%.

Data from the neuropathologically confirmed group were used to generate the Kaplan–Meier curves for each APOE genotype that are shown in Fig. 1. The curves indicate the percentage of neuropathologically confirmed cases and controls who remained free from Alzheimer’s dementia as a function of age. When estimated ages at dementia onset were not available, ages at death were used as a proxy.

CERAD scores and Braak stages were quantified for each *APOE* genotype in the aggregate group of neuropathologically confirmed cases and controls. Linear regression using the GLM model was used to assess the effect of each *APOE* genotype comparing to the reference genotype for CERAD and Braak measurements as quantitative outcomes with or without age at death and sex as covariates, repeated using CERAD scores as a covariate to assess residual effects of Braak stage.

### Reporting summary

Further information on research design is available in the Nature Research Reporting Summary linked to this article.

## Supplementary information

Supplementary Information

Reporting Summary

## Data Availability

APOE genotype and clinical/neuropathological phenotype data from ACT, ANDI, and ROS-MAP studies are accessible by directly applying to their study websites. APOE genotype and clinical/neuropathological phenotype data from other studies can be accessed by applying directly to The National Institute on Aging Genetics of Alzheimer’s Disease Data Storage Site (NIAGADS)—an NIA/NIH-sanctioned qualified-access data repository, under accession NG00075. The data that support the findings of this study are available from the NIAGAD website (https://www.niagads.org/).

## Code availability

The custom codes in R that generated the findings of this study are available by authors upon request.
