# Supplementary material for: Exceptionally low likelihood of Alzheimer’s dementia in APOE2 homozygotes from a 5,000-person neuropathological study
Source: Nat Commun. 2020 Feb 3;11:667. doi: 10.1038/s41467-019-14279-8 (PMC6997393; doi:10.1038/s41467-019-14279-8)
Supplement: Supplementary file 1 — Supplementary Information [file 41467_2019_14279_MOESM1_ESM.pdf]

SUPPLEMENTARY INFORMATION

Exceptionally Low Likelihood of Alzheimer's Dementia in APOE2 Homozygotes from a 5,000-Person Neuropathological Study

Eric M. Reiman[1,2,3,*], Joseph F. Arboleda-Velasquez[4], Yakeel T. Quiroz[5], Matthew J. Huentelman[6], Thomas G. Beach[1], Richard J. Caselli[7], Yinghua Chen[1], Yi Su[1], Amanda J. Myers[8], John Hardy[9], Jean Paul Vonsattel[10], Steven G. Younkin[11], David A. Bennett[12], Philip L. De Jager[13], Eric B. Larson[14], Paul K. Crane[15], C. Dirk Keene[16], M. Ilyas Kamboh[17], Julia K. Kofler[18], Linda Duque[19], John R. Gilbert[20], Harry E. Gwirtsman[21], Joseph D. Buxbaum[22], Dennis W. Dickson[23], Matthew P. Frosch[24], Bernardino Ghetti[25], Kathryn L. Lunetta[26], Li-San Wang[27], Bradley T. Hyman[28], Walter A. Kukull[29], Tatiana Foroud[30], Jonathan L. Haines[31], Richard P. Mayeux[32], Margaret A. Pericak-Vance[20], Julie A. Schneider[12], John Q. Trojanowski[27], Lindsay A. Farrer[26,33-35], Gerard D. Schellenberg[27], Gary W. Beecham[20], Thomas J. Montine[36], and Gyungah R. Jun[33] for the Alzheimer's Disease Genetics Consortium

Supplementary Table 1. Number of Clinically Diagnosed Alzheimer’s Dementia Cases and Controls for Each *APOE* Genotype

APOE	Neuropathologically Confirmed				Neuropathologically Unconfirmed				Combined			
	Cases	Controls	Total	%	Cases	Controls	Total	%	Cases	Controls	Total	%
2/2	5	19	24	0.5	16	70	86	0.4	21	89	110	0.4
2/3	113	147	260	5.2	450	1,610	2060	8.6	563	1,757	2320	8.0
3/3	1,273	638	1911	38.2	3,604	8,175	11779	49.4	4,877	8,813	13690	47.4
2/4	107	20	127	2.54	311	286	597	2.5	418	306	724	2.5
3/4	1,897	155	2052	41.0	4,690	2,998	7688	32.2	6,587	3,153	9740	33.7
4/4	623	10	633	12.6	288	1,359	1647	6.9	1,982	2,98	1982	6.9
TOTAL	4,018	989	5007	100	10,430	13,427	23857	100	14,448	14,416	28864	100

This table provides a summary of all cases with the clinical diagnosis of Alzheimer’s dementia and all cognitively unimpaired controls with APOE genotypes in the ADGC database. Participants in the combined group consisted of neuropathologically confirmed autopsy and neuropathologically unconfirmed clinical subjects, excluding neuropathologically misclassified subjects. Since the ADGC used both clinical and neuropathological criteria to prioritize autopsy participants in the ADGC database, the table does not include a substantial number of clinically characterized but neuropathologically mischaracterized cases and controls.

Supplementary Table 2. Characteristics of Alzheimer's Dementia Cases and Controls

APOE	Neuropathologically Confirmed					Neuropathologically Unconfirmed				
	AD Cases			Controls		AD Cases			Controls	
	AAO	AAD	% Female	AAD	% Female	AAO	AAE	% Female	AAE	% Female
2/2	74.3 ± 0.8	82.3 ± 7.3	40	85.2 ± 9.0	50	79.3 ± 8.2	81.6 ± 8.4	0.63	77.4 ± 8.2	50
2/3	79.4 ± 9.0	86.3 ± 8.8	60	83.5 ± 8.4	56	77.2 ± 8.4	80.8 ± 8.1	0.54	77.3 ± 8.0	59
3/3	77.1 ± 8.9	83.9 ± 8.2	62	82.1 ± 9.0	51	76.3 ± 7.8	80.6 ± 7.6	0.59	76.6 ± 7.9	60
2/4	74.0 ± 6.1	83.3 ± 7.1	68	82.5 ± 7.8	58	75.2 ± 6.9	79.9 ± 7.1	0.63	75.4 ± 7.5	58
3/4	73.3 ± 7.2	81.9 ± 7.2	56	80.9 ± 8.1	46	73.3 ± 6.7	78.4 ± 6.7	0.61	74.4 ± 7.6	61
4/4	69.9 ± 6.1	79.0 ± 6.5	53	79.0 ± 7.6	50	69.5 ± 5.9	75.3 ± 6.7	0.55	72.2 ± 6.7	62
Total	74.2 ± 8.0	82.3 ± 7.7	58	82.1 ± 8.7	51	74.1 ± 7.4	74.0 ± 7.0	0.59	76.1 ± 7.9	60

The neuropathologically confirmed group contained participants available with clinical diagnosis, the APOE genotype, and an age variable (age at dementia onset, age at death, or age at last clinical evaluation). Affection status of cases and controls in the autopsied sample was defined by clinical diagnosis.

The neuropathologically unconfirmed group includes clinically diagnosed but neuropathologically uncharacterized cases and controls.

AAO: Estimated age at dementia onset (when available) in the Alzheimer's dementia cases in years.

Only two APOE2 homozygotes with neuropathologically confirmed Alzheimer's dementia had an estimated AAO. AAO in the 65 combined APOE2/2 and 2/3 groups with neuropathologically confirmed Alzheimer's dementia was 79.3±9.0 years.

AAD: Age at death in the neuropathologically confirmed cases and controls in years.

AAE: Age at last clinical evaluation (when available) in the combined cases and controls in years.

Mean (Mean) and standard deviation (SD) for AAO, AAD, and AAE ranged from Mean-SD and Mean+SD, Mean±SD.

Supplementary Table 3. Alzheimer's Dementia Odds Ratios for Each APOE genotype after Adjustment for Age and Sex

APOE	Neuropathologically Confirmed			Neuropathologically Unconfirmed		
	OR	95% CI	P	OR	95% CI	P
Genotype						
2/2	0.16	0.06 - 0.43	$3.0x10^{-4}$	0.52	0.3 - 0.89	0.018
2/3	0.40	0.31 - 0.53	$3.6x10^{-11}$	0.63	0.54 - 0.75	$8.6x10^{-8}$
2/4	2.47	1.51 - 4.04	$3.4x10^{-4}$	2.49	2.05 - 3.04	$1.2x10^{-19}$
3/4	5.71	4.71 - 6.92	$8.2x10^{-68}$	3.55	3.19 - 3.96	$2.0x10^{-115}$
4/4	26.93	14.39 - 50.38	$2.1x10^{-24}$	10.94	9.29 - 12.89	$3.2x10^{-180}$
Allelic Dose						
2	0.40	0.32 - 0.51	$1.0x10^{-13}$	0.64	0.58 - 0.72	$1.7x10^{-15}$
4	5.57	4.69 - 6.61	$1.3x10^{-81}$	3.42	3.24 - 3.60	$<10^{-300}$

For genotypic association tests, odds ratio (OR), 95% confidence interval (CI), and P value (P) for each APOE genotype compared to the APOE3/3 genotype were calculated under a logistic regression model.

For allelic association tests, OR, CI, and P associated with APOE2 allelic dose in APOE4 non-carriers (APOE2/2<2/3<3/3) and APOE4 allelic dose in APOE2 non-carriers (APOE4/4>3/4>3/3) in an additive genetic model were generated under a logistic regression model.

Supplementary Table 4. Alzheimer's Dementia Odds Ratios for Each APOE Genotype in the Combined Group

APOE	Model 1 no adjustment			Model 2 age and sex adjustment			Model 3 age, sex and autopsy adjustment		
	OR	95% CI	P	OR	95% CI	P	OR	95% CI	P
Genotype									
2/2	0.43	0.26 - 0.70	$7.4x10^{-4}$	0.42	0.26 - 0.7	$7.1x10^{-4}$	0.35	0.20 - 0.61	$2.0x10^{-4}$
2/3	0.58	0.52 - 0.64	$1.9x10^{-25}$	0.58	0.52 - 0.64	$3.1x10^{-25}$	0.59	0.53 - 0.66	$1.1x10^{-21}$
2/4	2.47	2.12 - 2.87	$2.7x10^{-31}$	2.49	2.14 - 2.90	$9.8x10^{-32}$	2.49	2.13 - 2.92	$3.8x10^{-30}$
3/4	3.78	3.57 – 4.00	$<10^{-300}$	3.78	3.57 – 4.00	$<10^{-300}$	3.71	3.49 - 3.94	$<10^{-300}$
4/4	12.02	10.58 - 13.66	$<10^{-300}$	12.28	10.76 - 14.01	$<10^{-300}$	11.39	9.96 - 13.02	$3.1x10^{-277}$
Allelic Dose									
2	0.59	0.53 - 0.65	$9.1x10^{-27}$	0.59	0.53 - 0.65	$1.4x10^{-26}$	0.59	0.53 - 0.65	$3.1x10^{-24}$
4	3.65	3.48 - 3.82	$<10^{-300}$	3.64	3.47 - 3.83	$<10^{-300}$	3.55	3.38 - 3.74	$<10^{-300}$

The combined group included the 28,864 cases and controls from the neuropathologically confirmed and unconfirmed groups.

Association tests in a logistic regression model were conducted using clinical diagnosis as an outcome without adjustment (Model 1), with age and sex as covariates (Model 2), and with age, sex, and autopsy status as covariates (Model 3).

For genotypic association tests, odds ratio (OR), 95% confidence interval (CI), and P value (P) for each APOE genotype compared to the APOE3/3 genotype were calculated under a logistic regression model.

For allelic association tests, OR, CI, and P associated with APOE2 allelic dose in APOE4 non-carriers (APOE2/2<2/3<3/3) and APOE4 allelic dose in APOE2 non-carriers (APOE4/4>3/4>3/3) in an additive genetic model were generated under a logistic regression model.

Supplementary Table 5. Number of Other Neuropathological Diagnoses for Each APOE Genotype

APOE	Congophilic Amyloid Angiopathy (CAA)		Lewy Body Disease (LBD)		Vascular Brain Injury (VBI)		Hippocampal Sclerosis (HS)	
	with	without	with	without	with	without	with	without
2/2	2	6	3	11	2	5	0	11
2/3	56	68	43	167	75	68	14	142
3/3	454	563	342	1054	482	583	104	1031
2/4	51	22	30	36	33	44	8	66
3/4	792	260	501	896	419	755	137	1058
4/4	292	71	156	252	112	232	27	317
TOTAL	1647	1090	1079	2428	1123	1687	290	2625

Supplementary Table 6. Odds Ratios for Other Assessed Neuropathological Diagnoses

APOE	CAA			LBD			VBI			HS		
	OR	95% CI	P	OR	95% CI	P	OR	95% CI	P	OR	95% CI	P
Unadjusted												
2/2	0.41	0.08 - 2.06	0.28	0.84	0.23 - 3.03	0.79	0.48	0.09 - 2.51	0.39	NC	NC	NC
2/3	1.02	0.7 - 1.49	0.91	0.79	0.56 - 1.13	0.20	1.33	0.94 - 1.89	0.11	0.98	0.54 - 1.76	0.94
2/4	2.87	1.72 - 4.81	$6.3x10^{-5}$	2.18	1.38 - 3.45	$8.2x10^{-4}$	0.91	0.57 - 1.45	0.68	1.20	0.56 - 2.57	0.64
3/4	2.73	2.29 - 3.25	$2.3x10^{-28}$	1.72	1.46 - 2.03	$8.9x10^{-11}$	0.67	0.57 - 0.8	$4.5x10^{-6}$	1.28	0.98 - 1.68	0.07
4/4	5.10	3.83 - 6.8	$1.6x10^{-27}$	1.91	1.51 - 2.41	$7.2x10^{-8}$	0.58	0.45 - 0.75	$4.0x10^{-5}$	0.84	0.54 - 1.31	0.45
Adjusted for age and sex												
2/2	0.42	0.08 - 2.1	0.29	0.86	0.23 - 3.12	0.81	0.50	0.09 - 2.74	0.43	NC	NC	NC
2/3	1.12	0.76 - 1.63	0.57	0.84	0.59 - 1.21	0.34	1.15	0.8 - 1.65	0.45	0.93	0.51 - 1.67	0.80
2/4	2.65	1.57 - 4.45	$2.5x10^{-4}$	2.11	1.33 - 3.35	$1.6x10^{-3}$	1.09	0.68 - 1.77	0.72	1.27	0.59 - 2.74	0.55
3/4	2.52	2.1 - 3.02	$3.6x10^{-23}$	1.55	1.31 - 1.83	$3.8x10^{-7}$	0.86	0.72 - 1.04	0.12	1.54	1.16 - 2.04	$3.1x10^{-3}$
4/4	4.33	3.2 - 5.87	$1.1x10^{-20}$	1.56	1.22 - 2.01	$4.7x10^{-4}$	0.93	0.7 - 1.23	0.61	0.96	0.59 - 1.55	0.86
Adjusted for age, sex, and neuropathological diagnosis of AD												
2/2	0.95	0.16 - 5.55	0.96	1.67	0.43 - 6.49	0.46	0.67	0.12 - 3.71	0.65	NC	NC	NC
2/3	1.49	0.98 - 2.26	0.06	1.02	0.7 - 1.48	0.92	1.23	0.85 - 1.77	0.27	1.08	0.59 - 1.96	0.80
2/4	2.52	1.46 - 4.36	$9.5x10^{-4}$	1.88	1.17 - 3.02	$9.4x10^{-3}$	1.05	0.65 - 1.71	0.83	1.16	0.54 - 2.52	0.70
3/4	2.02	1.67 - 2.45	$4.7x10^{-13}$	1.25	1.05 - 1.49	0.01	0.80	0.66 - 0.96	0.02	1.31	0.99 - 1.75	0.06
4/4	3.34	2.45 - 4.55	$5.2x10^{-14}$	1.20	0.92 - 1.55	0.18	0.86	0.64 - 1.14	0.29	0.83	0.51 - 1.34	0.45

CAA: congophilic amyloid angiopathy; LBD: Lewy body disease; VBI: vascular brain injury; HS: hippocampal sclerosis.

Statistical tests for APOE2/2 and APOE4/4 genotypes compared to the APOE3/3 genotype were not converged (NC) by the extremely small number of persons with and without CAA, LBD, VBI, and HS.

Since TDP-43 pathology and microinfarcts were not characterized in many of the participants, the impact of APOE genotypes on the presence or absence of those pathological diagnoses were not assessed.

Supplementary Table 7. Other Neuropathological Diagnosis Odds Ratios with APOE2 and APOE4 Allelic Doses

Model and Diagnosis	APOE2 Allelic Dose			APOE4 Allelic Dose		
	OR	95% CI	P	OR	95% CI	P
Unadjusted						
CAA	0.93	0.67 - 1.31	0.68	2.43	2.13 - 2.76	$1.1x10^{-39}$
LBD	0.82	0.60 - 1.13	0.22	1.46	1.31 - 1.62	$1.4x10^{-11}$
VBI	1.20	0.87 - 1.65	0.27	0.73	0.65 - 0.83	$3.3x10^{-7}$
HS	0.85	0.49 - 1.48	0.58	1.02	0.85 - 1.23	0.83
Adjusted for age and sex						
CAA	1.00	0.71 - 1.41	0.99	2.26	1.97 - 2.59	$1.9x10^{-31}$
LBD	0.86	0.62 - 1.19	0.36	1.32	1.17 - 1.47	$2.6x10^{-6}$
VBI	1.06	0.76 - 1.48	0.71	0.94	0.82 - 1.06	0.31
HS	0.82	0.47 - 1.42	0.47	1.17	0.96 - 1.43	0.12
Adjusted for age, sex, and neuropathological diagnosis of AD						
CAA	1.36	0.94 - 1.98	0.11	1.90	1.66 - 2.19	$2.1x10^{-19}$
LBD	1.07	0.76 - 1.50	0.71	1.13	1.01 - 1.28	0.04
VBI	1.16	0.83 - 1.62	0.39	0.89	0.78 - 1.02	0.09
HS	0.98	0.55 - 1.73	0.95	1.05	0.86 - 1.29	0.61

CAA: congophilic amyloid angiopathy..LBD: Lewy body disease; VBI: vascular brain injury; HS: hippocampal sclerosis.

Odds ratio (ORs), 95% confidence interval (CI), and P value (P) associated with APOE2 allelic dose in APOE4 non-carriers (APOE2/2<2/3<3/3) and APOE4 allelic dose in APOE2 non-carriers (APOE4/4>3/4>3/3) were generated using allelic association tests with an additive genetic model.

Since TDP-43 pathology and microinfarcts were not assessed in all participants, the impact of APOE2 and APOE4 allelic doses on the presence or absence of those diagnoses were not assessed

Supplementary Table 8. Summary and Association of CERAD Scores and Braak Stages

APOE	CERAD (Neuritic Aβ Plaque) Score						Braak (Neurofibrillary Tangle [PHF Tau]) Stage					
	N	Mean±SD	Unadjusted		Adjusted for AAD and Sex		N	Mean±SD	Unadjusted		Adjusted for AAD and Sex	
			BETA±SE	P	BETA±SE	P			BETA±SE	P	BETA±SE	P
2/2	24	0.94±1.14	-1.04±0.30	$5.6x10^{-4}$	-1.51±0.44	6.3E-04	16	2.38±1.71	-1.65±0.45	$2.7x10^{-4}$	-1.51±0.44	$6.3x10^{-4}$
2/3	260	1.35±1.32	-0.63±0.09	$1.9x10^{-11}$	-0.78±0.13	1.3E-09	227	3.17±1.95	-0.86±0.13	$4.2x10^{-11}$	-0.78±0.13	$1.3x10^{-9}$
3/3	1911	1.98±1.24	Ref	Ref	Ref	Ref	1583	4.03±1.80	Ref	Ref	Ref	Ref
2/4	127	2.40±0.99	0.42±0.14	$1.9x10^{-3}$	0.84±0.05	1.5E-53	107	4.60±1.50	0.57±0.18	$1.4x10^{-3}$	0.46±0.17	$8.8x10^{-3}$
3/4	2052	2.68±0.72	0.70±0.04	$4.1x10^{-76}$	1.08±0.08	5.2E-37	1763	4.96±1.26	0.93±0.05	$1.7x10^{-65}$	0.84±0.05	$1.5x10^{-53}$
4/4	549	2.88±0.42	0.90±0.06	$7.8x10^{-51}$	-1.51±0.44	6.3E-04	633	5.28±0.93	1.25±0.08	$1.1x10^{-51}$	1.08±0.08	$5.2x10^{-37}$

Aβ: amyloid-β; PHF: paired helical filament; AAD: Age at Death; AAD: age at death. CERAD Scores reflect the density of neuritic plaques: 0 (none), 1 (sparse), 2 (moderate), and 3 (frequent); Braak Stages reflect the spatial distribution of neurofibrillary tangles: 0 (none), I-II (transentorhinal and entorhinal cortex), III-IV (hippocampal and neighboring limbic areas), and V-VI (neocortical areas).

Ref: reference genotype to test association of each *APOE* genotype in a linear regression model Mean (Mean) and standard deviation (SD) for AAO, AAD, and AAE ranged from Mean-SD and Mean+SD, Mean±SD.

Beta estimate (BETA), standard error (SE), and P value (P) for each *APOE* genotype compared to the APOE3/3 genotype as a reference (ref) were calculated under a linear regression model.

Unadjusted: results from a linear regression for CERAD scores or Braak stages without adjusting for any covariates.

Adjusted for AAD and Sex: results from a linear regression for CERAD scores or Braak stages after adjusting for age at death (AAD) and sex as covariates.

Supplementary Figure 1. Impact of APOE2 and APOE4 Allelic Doses in the Combined Group.

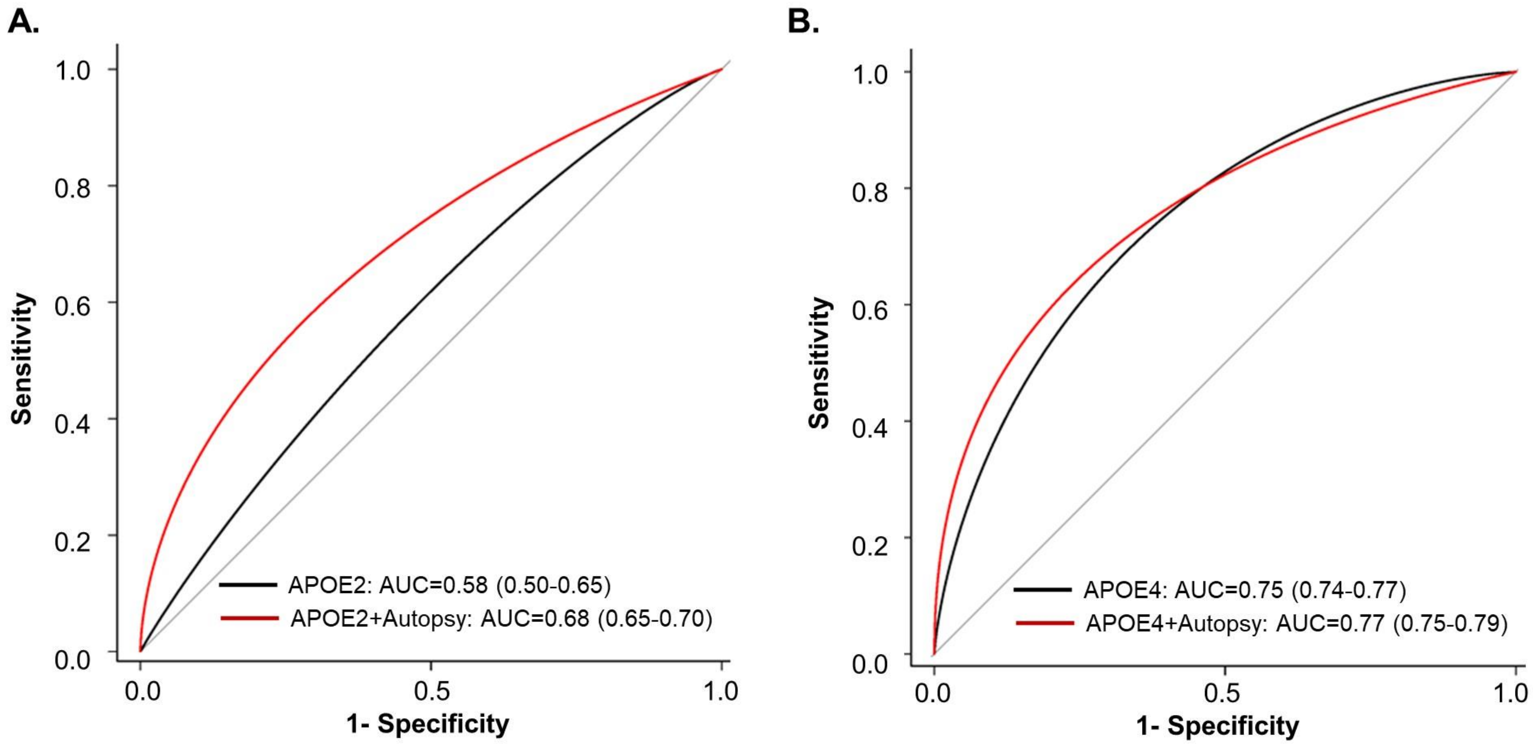

Receiver Operating Characteristic (ROC) curves reflect the impact of APOE2 allelic dose **(A)** and APOE4 allelic dose **(B)** on the sensitivity and specificity to classify clinically diagnosed Alzheimer's dementia cases and controls. ROC curves in the neuropathologically confirmed autopsy group were shown in red and those in the neuropathologically unconfirmed non-autopsy group are shown in black. As reflected by the Area Under the Curve (AUC), an indicator of classification accuracy, and their 95% confidence intervals, APOE2 and APOE4 allelic doses were each associated with significantly higher AUCs in the neuropathologically confirmed and unconfirmed groups; APOE2 allelic dose was associated with a significantly greater AUC in the neuropathologically confirmed than unconfirmed group, whereas APOE4 allelic dose was not**.**

Supplementary Figure 2. CERAD Scores and Braak Stages for Each APOE Genotype in the Neuropathologically Confirmed Group.

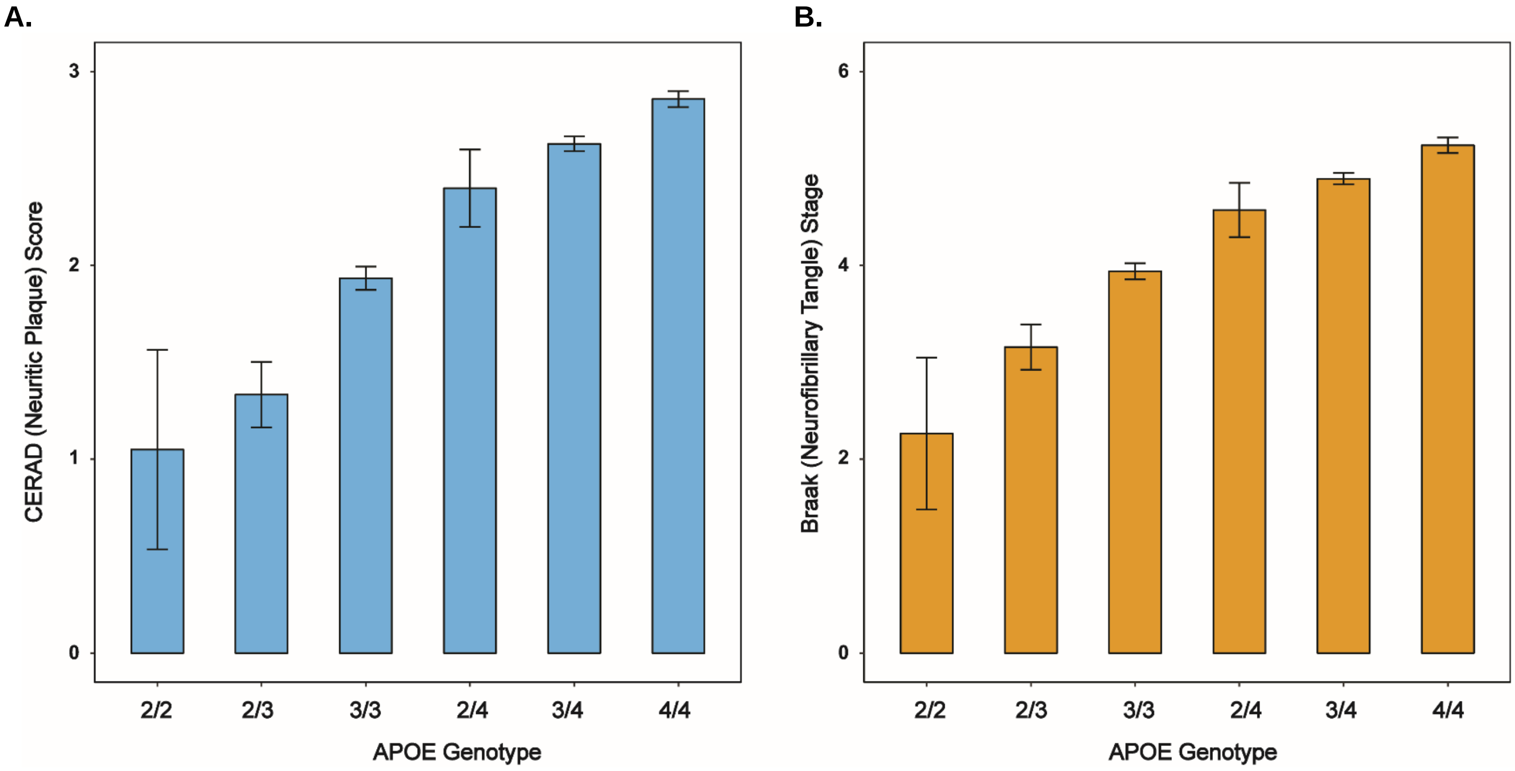

The means (mid-points) and standard deviations from the means (error bars) from CERAD (Neuritic Aβ Plaque) scores **(A)** and Braak (Tau Tangle) stages **(B)** for each APOE genotype in Supplementary Table 8 were shown as the bar graphs.
