# Supplementary material for: Exceptionally low likelihood of Alzheimer’s dementia in APOE2 homozygotes from a 5,000-person neuropathological study
Source: Nat Commun. 2020 Feb 3;11:667. doi: 10.1038/s41467-019-14279-8 (PMC6997393; doi:10.1038/s41467-019-14279-8)
Supplement: Supplementary file 2 — Reporting Summary [file 41467_2019_14279_MOESM2_ESM.pdf]

nature research

Corresponding author(s): *Double-blind peer review submissions: write DBPR and your manuscript number here instead of author names.*

Last updated by author(s): *YYYY-MM-DD*

Reporting Summary

Nature Research wishes to improve the reproducibility of the work that we publish. This form provides structure for consistency and transparency in reporting. For further information on Nature Research policies, see Authors & Referees and the Editorial Policy Checklist.

Statistics

For all statistical analyses, confirm that the following items are present in the figure legend, table legend, main text, or Methods section.

n/a	Confirmed	
☐	☒	The exact sample size (*n*) for each experimental group/condition, given as a discrete number and unit of measurement
☒	☐	A statement on whether measurements were taken from distinct samples or whether the same sample was measured repeatedly
☒	☐	The statistical test(s) used AND whether they are one- or two-sided<br>*Only common tests should be described solely by name; describe more complex techniques in the Methods section.*
☐	☒	A description of all covariates tested
☐	☒	A description of any assumptions or corrections, such as tests of normality and adjustment for multiple comparisons
☐	☒	A full description of the statistical parameters including central tendency (e.g. means) or other basic estimates (e.g. regression coefficient) AND variation (e.g. standard deviation) or associated estimates of uncertainty (e.g. confidence intervals)
☐	☒	For null hypothesis testing, the test statistic (e.g. *F*, *t*, *r*) with confidence intervals, effect sizes, degrees of freedom and *P* value noted<br>*Give P values as exact values whenever suitable.*
☒	☐	For Bayesian analysis, information on the choice of priors and Markov chain Monte Carlo settings
☒	☐	For hierarchical and complex designs, identification of the appropriate level for tests and full reporting of outcomes
☒	☐	Estimates of effect sizes (e.g. Cohen's *d*, Pearson's *r*), indicating how they were calculated

Our web collection on statistics for biologists contains articles on many of the points above.

Software and code

Policy information about availability of computer code

Data collection

no software was used. Data has been previously collected in each study from Alzheimer's Disease Genetics Consortium

Data analysis

RStudio Version 1.1.456.

```
###########
# function
###########
main.gee.test <- function(trait,subgroup,outcome,apoe_colname,form)
{
  temp <- trait[!is.na(trait[,outcome]) & !is.na(trait[,apoe_colname]), c(id_colname, status_clinic, neuro_colname, apoe_colname, 
age_colname, sex_colname, aao_colname)]
  mydata <- data.frame(temp)
  colnames(mydata) <- c(id_colname, status_clinic, neuro_colname, apoe_colname, age_colname, sex_colname, aao_colname)
  gee.result <- matrix(NA,nrow=5,ncol=3)

  # run gee treating as family
  print(paste(outcome,subgroup,"gee",sep=" "))
  gee.coef <- try(summary(gee(formula = as.formula(form),data = mydata,
                             id = as.numeric(as.factor(mydata[,id_colname])),
                             family = binomial(link="logit"),  corstr = "independence"))$coef, silent=T)
  if(!"try-error" %in% class(gee.coef)) {
    mygee <- unlist(gee.coef)
    print(mygee)
    print(pnorm(-abs(mygee[,5]))*2)
    #result <-
```

```
paste(subgroup,outcome,analysis.method,analysis.adjust,alt,ref,myresult[1],myresult[2],format(myresult[4],scientific=T,digits=4),sep="\t
")
  } else {
    error <- paste("gee->error:",subgroup,outcome," ")
    print(error)
    #result <- paste(subgroup,outcome,analysis.method,analysis.adjust,alt,ref,NA,NA,NA,sep="\t")
  }
}

main.glm.test <- function(trait,subgroup,outcome,apoe_colname,form)
{
  temp <- trait[!is.na(trait[,outcome]), c(id_colname, status_clinic, neuro_colname,apoe_colname, age_colname, sex_colname,
aao_colname)]
  mydata <- data.frame(temp)
  colnames(mydata) <- c(id_colname, status_clinic, neuro_colname, apoe_colname, age_colname, sex_colname, aao_colname)

  # run glm
  print(paste(outcome,subgroup,"glm",sep=" "))
  glm.obj <- glm(formula = as.formula(form),data = mydata,family=quasibinomial(link="logit"))
  glm.result <- summary(glm.obj)$coefficients
  print(glm.result)
  #print(format(glm.result,scientific=T,digits=4))
}

##############################################
# function: apoe subgroup analysis
# return: apoe analysis results
##############################################
apoe.gee.analysis <- function(file.name,trait,subgroup,outcome,analysis.method,analysis.adjust,apoe_colname,form)
{
  result <-  ""
  for (i in 1:length(refs))
  {
    curef <- refs[i]
    curef.val <- refvals[i]

    for(model in names(group)) {
      if (model != curef) {
        temp <- trait[!is.na(trait[,outcome]), c(id_colname, status_clinic, neuro_colname, apoe_colname, age_colname, sex_colname,
aao_colname)]
        mydata <- data.frame(temp[(temp[,apoe_colname] %in% group[[model]]) | (temp[,apoe_colname] %in% group[[curef]]),
c(id_colname, status_clinic, neuro_colname, apoe_colname, age_colname, sex_colname, aao_colname)])
        colnames(mydata) <- c(id_colname, status_clinic, neuro_colname, apoe_colname, age_colname, sex_colname, aao_colname)
        mydata[,apoe_colname]   <- ifelse(mydata[,apoe_colname] == curef.val,0,1)

        alt  <- model
        ref  <- curef

        # run gee treating as family
        gee.coef <- try(summary(gee(formula = as.formula(form),data = mydata,
                              id = as.numeric(as.factor(mydata[,id_colname])),
                              family = binomial(link="logit"),  corstr = "independence"))$coef, silent=T)
        if(!"try-error" %in% class(gee.coef)) {
          mygee <- unlist(gee.coef)
          myresult <- pnorm(-abs(mygee[,5]))*2
          result <-
paste(subgroup,outcome,analysis.method,analysis.adjust,alt,ref,mygee[2,1],mygee[2,4],format(myresult[2],scientific=T,digits=4),sep="\t"
)
        } else {
          error <- paste("gee->error:",subgroup,outcome,analysis.method,analysis.adjust,alt,ref," ")
          print(error)
          result <- paste(subgroup,outcome,analysis.method,analysis.adjust,alt,ref,NA,NA,NA,sep="\t")
        }

        write(result,file=outfile,ncolumns=1,append=T)

      } #if-model==ref
    } #inner-for each apoe genotype
  } #outer-for each apoe reference
}

apoe.glm.analysis <- function(file.name,trait,subgroup,outcome,analysis.method,analysis.adjust,apoe_colname,form)
{
  result <-  ""
  for (i in 1:length(refs))
```

```
{
  curef <- refs[i]
  curef.val <- refvals[i]

  for(model in names(group)) {
    if (model != curef) {
      temp <- trait[!is.na(trait[,outcome]), c(id_colname, status_clinic, neuro_colname, apoe_colname, age_colname, sex_colname,
aao_colname)]
      mydata <- data.frame(temp[(temp[,apoe_colname] %in% group[[model]]) | (temp[,apoe_colname] %in% group[[curef]]),
c(id_colname, status_clinic, neuro_colname, apoe_colname, age_colname, sex_colname, aao_colname)])
      colnames(mydata) <- c(id_colname, status_clinic, neuro_colname, apoe_colname, age_colname, sex_colname, aao_colname)
      mydata[,apoe_colname]   <- ifelse(mydata[,apoe_colname] == curef.val,0,1)

      alt  <- model
      ref  <- curef

      # run glm
      glm.obj <- glm(formula = as.formula(form),data = mydata,family=quasibinomial(link="logit"))
      myresult <- summary(glm.obj)$coefficients

      # create result
      result <-
paste(subgroup,outcome,analysis.method,analysis.adjust,alt,ref,myresult[2,1],myresult[2,2],format(myresult[2,4],scientific=T,digits=4),se
p="\t")
      write(result,file=outfile,ncolumns=1,append=T)

    } #if-model==ref
  } #inner-for each apoe genotype
 } #outer-for each apoe reference
}
```

For manuscripts utilizing custom algorithms or software that are central to the research but not yet described in published literature, software must be made available to editors/reviewers. We strongly encourage code deposition in a community repository (e.g. GitHub). See the Nature Research guidelines for submitting code & software for further information.

Data

Policy information about availability of data

All manuscripts must include a data availability statement. This statement should provide the following information, where applicable:
- Accession codes, unique identifiers, or web links for publicly available datasets
- A list of figures that have associated raw data
- A description of any restrictions on data availability

The data that support the findings of this study are available from the NIAGAD website (https://www.niagads.org/).

Field-specific reporting

Please select the one below that is the best fit for your research. If you are not sure, read the appropriate sections before making your selection.

☒ Life sciences ☐ Behavioural & social sciences ☐ Ecological, evolutionary & environmental sciences

For a reference copy of the document with all sections, see nature.com/documents/nr-reporting-summary-flat.pdf

Life sciences study design

All studies must disclose on these points even when the disclosure is negative.

Sample size	The total sample in this study was previously available as a combined dataset in the Alzheimer's Disease Genetics Consortium (ADGC).
Data exclusions	We conducted statistical analyses using different groups of subjects, the neuropathologically confirmed group excluding neuropathologically misclassified and unevaluated subjects, the clinical group excluding autopsied subjects, and the combined group without excluding any subjects. We compared effect sizes from the different groups to evaluate impact of APOE genotypes on Alzheimer's disease when diagnosis was validated both clinically and neuropathologically.
Replication	The findings of this study using the clinically diagnosed subjects have been previously validated. Since this study contains the largest collection of autopsied subjects, there are no other autopsied subjects with both extensively evaluated clinically and neuropathologically.
Randomization	We grouped participants based on autopsy status. We conducted statistical analysis controlling autopsy status as well as age and sex if these covariates affect on our findings.
Blinding	Data collection was previously and separately achieved. Group allocation was conducted during analysis stage by investigators independent from data collection.

Reporting for specific materials, systems and methods

We require information from authors about some types of materials, experimental systems and methods used in many studies. Here, indicate whether each material, system or method listed is relevant to your study. If you are not sure if a list item applies to your research, read the appropriate section before selecting a response.

Materials & experimental systems

n/a	Involved in the study
☒	☐ Antibodies
☒	☐ Eukaryotic cell lines
☒	☐ Palaeontology
☒	☐ Animals and other organisms
☐	☒ Human research participants
☒	☐ Clinical data

Methods

n/a	Involved in the study
☒	☐ ChIP-seq
☒	☐ Flow cytometry
☒	☐ MRI-based neuroimaging

Human research participants

Policy information about studies involving human research participants

Population characteristics	*Describe the covariate-relevant population characteristics of the human research participants (e.g. age, gender, genotypic information, past and current diagnosis and treatment categories). If you filled out the behavioural & social sciences study design questions and have nothing to add here, write "See above."*
Recruitment	Participants were recruited previously by each study in the Alzheimer's Disease Genetics Consortium (ADGC).
Ethics oversight	Alzheimer's Disease Genetics Consortium

Note that full information on the approval of the study protocol must also be provided in the manuscript.
